# Nutrient Removal Process and Cathodic Microbial Community Composition in Integrated Vertical-Flow Constructed Wetland – Microbial Fuel Cells Filled With Different Substrates

**DOI:** 10.3389/fmicb.2020.01896

**Published:** 2020-08-05

**Authors:** Fei Zhong, Chunmei Yu, Yanhong Chen, Xue Wu, Juan Wu, Guoyuan Liu, Jian Zhang, Zifa Deng, Shuiping Cheng

**Affiliations:** ^1^School of Life Sciences, Nantong University, Nantong, China; ^2^Key Laboratory of Yangtze River Water Environment, Ministry of Education, College of Environmental Science and Engineering, Tongji University, Shanghai, China; ^3^Shanghai Institute of Pollution Control and Ecological Security, Shanghai, China

**Keywords:** substrate selection, hydraulic retention time, nitrogen transformation, functional bacterial groups, bioelectrical signals

## Abstract

An integrated vertical-flow constructed wetland-microbial fuel cell system (CW-MFC), consisting of an up-flow chamber and a down-flow chamber, was constructed to treat synthetic sewage wastewater. The performance of CW-MFCs filled with different substrates [i.e., ceramsite (CM-A), quartz (CM-B), and zeolite (CM-C) granules] under various hydraulic retention times (HRTs, 7.6, 4.0, and 2.8 d) was evaluated. Efficient and stable nitrogen (N) and phosphorus (P) removals were observed in CM-A under different HRTs, while the voltage outputs of the CW-MFCs was greatly reduced as the HRTs decreased. With an HRT of 2.8 d, the ammonium (NH_4_^+^-N) and orthophosphate (PO_4_^3–^-P) removal efficiencies in CM-A were as high as 93.8 and 99.6%, respectively. Bacterial community analysis indicates that the N removal in the cathode area of CM-A could potentially benefit from the appearance of nitrifying bacteria (e.g., *Nitrosomonas* and *Nitrospira*) and relatively high abundance of denitrifiers involved in simultaneous nitrification and denitrification (e.g., *Hydrogenophaga*, *Zoogloea*, and *Dechloromonas*) and denitrifying sulfide removal (e.g., *Thauera*). Additionally, the difference in N removal efficiency among the CW-MFCs could be partly explained by higher iron (Fe) content in milled ceramsite granules and higher abundance of denitrifiers with nitrate reduction and ferrous ions oxidation capabilities in CM-A compared with that in CM-B and CM-C. Efficient PO_4_^3–^-P removal in CM-A was mainly ascribed to substrate adsorption and denitrifying phosphorus (P) removal. Concerning the substantial purification performance in CM-A, ceramsite granules could be used to improve the nutrient removal efficiency in integrated vertical-flow CW-MFC.

## Introduction

Water eutrophication caused by the enrichment of nitrogen (N) and phosphorus (P) has become a worldwide environmental problem in recent years. To protect surface water bodies from eutrophication, rigorous N and P discharge regulations have been established. This, in turn, encourages the development of novel technologies for wastewater treatment.

Constructed wetlands (CWs) have been widely accepted as a preferable alternative for wastewater treatment because of their low initial investment costs, easy operation and maintenance, and good landscape integration. Meanwhile, CWs are being upgraded continuously to meet increasingly strict water pollutant discharge standards. Recently, the integration of CWs with microbial fuel cells (i.e., constructed wetland-microbial fuel cell system, CW-MFCs) has attracted worldwide attention (Doherty et al., 2015). In general, the efficiency of power generation and wastewater treatment is the focus of CW-MFC research. Concerning research on power generation, great efforts have been made to increase power density (Shen et al., 2018; Xu et al., 2019), though the usefulness of CW-MFCs system as an electricity generating device in real scenario has not yet been well examined. For research on wastewater treatment, attention has been extended from chemical oxygen demand (COD) removal to N removal (Xu et al., 2018).

More specifically for electricity generation, past research paid great attention to up-flow CW-MFCs, because such system could minimize dissolved oxygen (DO) at the anode while ensuring maximum availability in the cathode region, maximizing the redox gradient to generate an electrical current (Corbella et al., 2014). However, under the up-flow pattern, N removal might be affected due to insufficient nitrification and denitrification at the anode and cathode, respectively. Nitrification should be accomplished before the effluent enters into the cathode area to facilitate cathodic denitrification when ammonium (NH_4_^+^) is the dominant N species in the influent. Oon et al. (2017) improved the nitrification efficiency by supplying artificial aeration in the cathode region. However, it was usually difficult to balance nitrification and denitrification processes with supplementary aeration, especially given the fact that a high effluent nitrate concentration was often observed.

Wu et al. (2017) successfully improved the TN removal efficiency in an up-flow CW-MFC with closely spaced electrodes by applying central aeration and effluent recirculation. The improvement of the TN removal was ascribed to a higher abundance of denitrifiers and anaerobic ammonium oxidation bacteria in the bottom layer of the system. By comparing open- and closed-circuit CW-MFCs (down-flow), Wang et al. (2016) found that MFCs could improve N removal in CWs when the dominant N species in the influent was nitrate. The analysis of the anodic bacterial community showed that the relative abundance of nitrate-reducing bacteria (*Dechloromonas*, *Desulfobulbus*, *Flavobacterium*, *Propionivibrio*, and *Geobacter*) had been significantly increased in the closed-circuit CW-MFCs. Compared with effluent recirculation, treating the effluent from an up-flow CW-MFC by a follow-up down-flow CW-MFC would be a promising alternative, as this combined system decreases energy demand. Liu et al. (2019) developed an integrated vertical-flow (down-flow and up-flow in sequence) CW-MFC in which the anode and cathode were placed in the bottom of the down-flow chamber and the top of the up-flow chamber, respectively. In this configuration, the organic matter was consumed in front of the anode area because of aerobic oxidation. The traditional N removal pathway (i.e., aerobic nitrification and heterotrophic denitrification) might have been mainly responsible for the N removed. The study conducted by Liu et al. (2019) suggests that this type of integrated vertical-flow CW-MFC would be suitable for the treatment of high strength wastewater (e.g., swine wastewater). However, for wastewater with relatively low organic content, it is necessary to evaluate the performance of integrated vertical-flow CW-MFC consisting of an up-flow chamber and a down-flow chamber in sequence.

As an indispensable part of CW-MFC, substrate plays an essential role in N and P removal. Substrates (e.g., zeolite and ceramsite) with larger surface area and porous structure can improve the N and P adsorption and promote the development of biofilm (Wang et al., 2020). Yakar et al. (2018) investigated the effect of various types of substrates (sand, zeolite and volcanic cinder) on the performance of CW-MFC. Their study recommended that zeolite could be used to increase N and P removal efficiencies and bioelectric production in up-flow CW-MFC. However, it remains unclear whether or not the higher N removal efficiency in CW-MFC filled with zeolite is due to the promotion of active microbial groups involved in N removal. Using a pyrite-based down-flow CW-MFC, Ge et al. (2020) found enhanced nitrate and nitrite (NO_X_^–^) removal via the intensification of the autotrophic denitrification process in the anode area. MFCs had been successfully operated using nitrate as a terminal electron acceptor in the cathode (Clauwaert et al., 2007). Wang et al. (2019) claimed that the MFC could reduce the dependence of denitrification on organic carbon sources in CWs, and the N removal in the CW-MFC under low influent COD/N ratios could be ascribed to bioelectrochemical denitrification with autotrophic denitrifying bacteria by accepting electrons from the cathode. The potential influence of substrates selection on N removal process in the cathode area of CW-MFCs is to be investigated.

This study compared the performance of integrated vertical-flow CW-MFCs (consisting of an up-flow and a down-flow chamber) filled with different substrates (i.e., ceramsite, quartz, and zeolite granules) treating synthetic sewage wastewater. It aims to examine the potential nutrient removal processes in this type of CW-MFC, and to explore the effects of substrate selection on the nutrient removal processes. Moreover, the composition of bacterial community in the cathode area was analyzed using 16S rRNA gene sequencing to investigate the possible pathways of N and P removal. This study could be beneficial to optimize the integrated vertical-flow CW-MFCs and encourage the development of novel CW-MFCs for use in reality.

## Materials and Methods

### Construction of Integrated Vertical-Flow CW-MFCs

**FIGURE 1:**
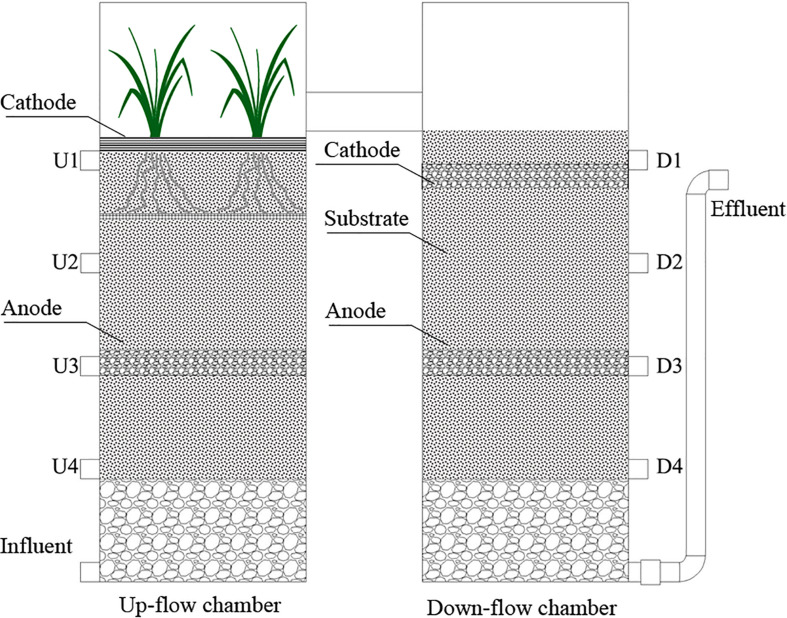
Schematic diagram of the integrated vertical-flow constructed wetland – microbial fuel cell systems. U4-U1 and D1-D4 are sampling ports located in the up-flow and down-flow chambers, respectively.

A schematic diagram of the integrated vertical flow CW-MFCs used in this study is shown in Figure 1. Along the water flow direction, each CW-MFC was separated into an up-flow chamber and a down-flow chamber. Both chambers were made of polyvinylchloride (PVC) tubes with dimensions of 120 mm in diameter and 350 mm in height, and were connected at the top. The total volume of each CW-MFC was 7.92 L, with a liquid volume of around 4.12 L.

In the up-flow chamber, *Canna indica* was planted, and an air-cathode made of carbon felt was placed at the water–air interface. Granular graphite (supported by a stainless-steel net) were used as the anode, and the electrodes were connected by an external electrical resistor of 1,000 Ω. In the down-flow chamber, both electrodes were made of granular graphite, with an external electrical resistor of 3,000 Ω used to connect them. The anode and cathode of the CW-MFCs were inoculated with anaerobic digested sludge and activated sludge, respectively. The sludges were collected from a wastewater treatment plant nearby. The voltage outputs of the three CW-MFCs were recorded with an automatic recorder (Wuhan YAV Electronic Technology Co., Ltd., China, Smart V). The power density was calculated by dividing the power with the working volume of the anode.

There were eight sampling ports in the CW-MFCs along the water flow direction [U4-U1 (up-flow chamber) and D1-D4 (down-flow chamber)]. The CW-MFCs were filled with different substrates, i.e., ceramsite (CM-A), quartz (CM-B), and zeolite (CM-C) granules with a diameter of 4–8 mm, which were the same as those reported by Zhong et al. (2015). Time replicates (*n* = 6) were used to compare the differences in purification performances among the three CW-MFCs filled with different substrates. During the experiment, the CW-MFCs were operated under different HRTs [i.e., 7.6 d (H1), 4.0 d (H2), and 2.8 d (H3)]. The CW-MFCs were operated in intermittent flow mode using self-starting pumps three times each day (i.e., at 6:00, 12:00, and 18:00). The pump running time (within 30 min) was set according to the hydraulic loading rates. The hydraulic loading rates were 0.54 L/d (H1), 1.03 L/d (H2), and 1.47 L/d (H3), respectively. The wetland systems were operated continuously for a period of around 120 days from June to October in 2018. Before the experiment, the CW-MFCs had been stabilized for 3 months. The composition of synthetic wastewater was similar to that reported by Villaseñor et al. (2013) with small changes. It was composed of glucose (160 mg/L), CH_3_COONa⋅3H_2_O (160 mg/L), NaHCO_3_ (111 mg/L), KH_2_PO_4_ (12 mg/L), K_2_HPO_4_ (40 mg/L), MgCl_2_⋅6H_2_O (37.1 mg/L), CaCl_2_⋅2H_2_O (30.1 mg/L), and NH_4_Cl (90 mg/L).

### Physicochemical Analysis

Water samples were collected weekly from the influent, effluent and eight sampling ports between 12:00 and 12:30. COD was determined using COD digestion vials (HACH, Loveland CO, 2125825) and a portable spectrophotometer (HACH, Loveland CO, DR2800). The concentrations of NH_4_^+^-N, NO_X_^–^-N, orthophosphate (PO_4_^3–^-P), sulfide (S^2–^-S), and sulfate (SO_4_^2–^-S) were measured using an automatic chemical analyzer (DeChem-Tech, Hamburg, CleverChem anna). DO and pH were measured with the Thermo Orion 5 Star portable meter (Thermo-Orion Inc., Waltham, MA, United States).

### Bacterial Community Analysis

The morphologies and elemental microanalysis of the original substrates and the substrates sampled at different layers of CW-MFCs were observed by scanning electron microscopy/energy dispersive X-ray spectrometry (SEM/EDS, Hitachi, Japan, S-3400N II) at the end of the experiment.

In each CW-MFC, three substrate samples were taken from the cathode area and mixed in polyethylene bags. A subsample was taken from each bag for bacterial community analysis. Any visible root or plant material was manually removed prior to homogenization. Samples were stored in a freezer at −80°C until Illumina sequencing analysis was conducted.

Genomic DNA was extracted from approximately 8 g of mixed substrate samples using an E.Z.N.A.^®^ soil DNA Kit (Omega Bio-tek, Norcross, GA, United States.) according to the manufacturer’s instructions. The DNA extract was checked on a 1% agarose gel, and the DNA concentration and purity were determined with a NanoDrop 2000 UV-vis spectrophotometer (Thermo Scientific, Wilmington, DE, United States). The hypervariable V3–V4 region of the bacterial 16S rRNA gene was amplified with the primer pair 338F (5′-ACTCCTACGGGAGGCAGCAG-3′) and 806R (5′-GGACTACHVGGGTWTCTAAT-3′) by an ABI GeneAmp^®^ 9700 PCR thermocycler (ABI, CA, United States). Purified amplicons were pooled in equimolar amounts and paired-end sequenced (2 × 300) on an Illumina MiSeq platform (Illumina, San Diego, CA, United States) according to the standard protocols by Majorbio Bio-Pharm Technology Co. Ltd. (Shanghai, China).

The raw 16S rRNA gene sequencing reads were demultiplexed, quality-filtered by Trimmomatic and merged by FLASH (Magoč and Salzberg, 2011). Operational taxonomic units (OTUs) with a 97% similarity cutoff were clustered using UPARSE^1^ (version 7.1), and chimeric sequences were identified and removed. The taxonomy of each OTU representative sequence was analyzed by RDP Classifier^2^ against the 16S rRNA database (e.g., Silva SSU128) using a confidence threshold of 0.7. Raw sequence data obtained was deposited in NCBI under the BioProject accession PRJNA629887.

### Statistical Analysis

All data are presented as the means ± SD (standard deviation); *n* refers to the number of the samples. Statistical analysis was performed using the SPSS 19.0 software package for Windows. Differences in effluent water quality among the CW-MFCs filled with different substrates and operated under different HRTs were evaluated using one-way ANOVA.

## Results

### Water Purification and Electricity Generation Performance

**FIGURE 2:**
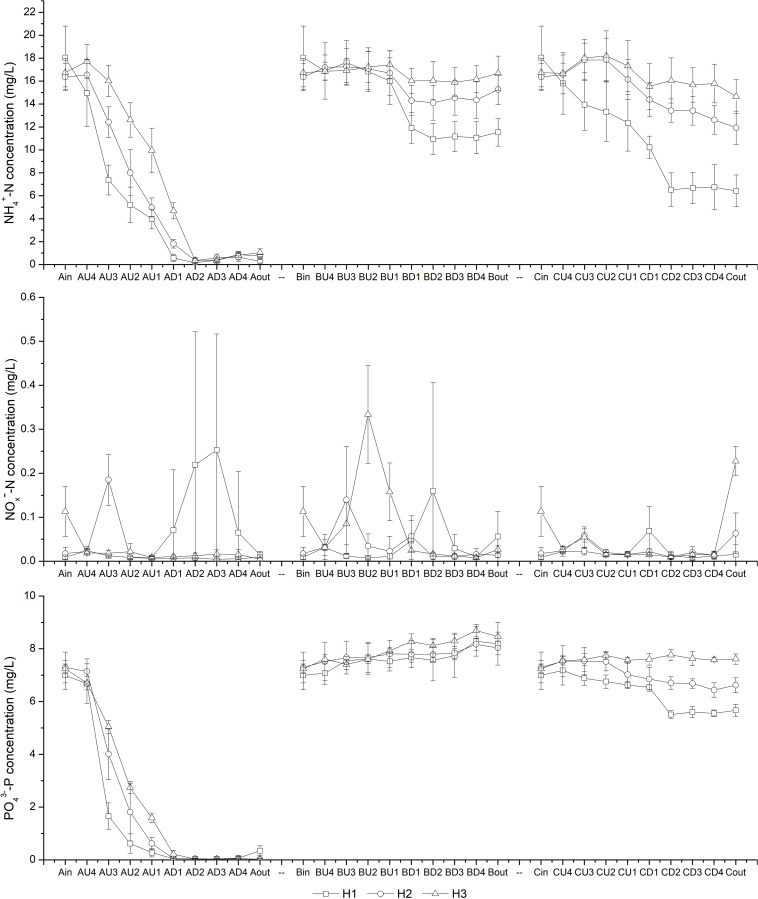
Variation in NH_4_^+^-N, NOx^–^-N, and PO_4_^3–^-P concentrations at different layers [influent (in), U4-U1, D1-D4 and effluent (out)] of CW-MFCs filled with ceramsite (CM-A), quartz (CM-B), and zeolite (CM-C) granules under various hydraulic retention times [7.6 d (H1), 4.0 d (H2), and 2.8 d (H3)].

The variations in N and P concentrations inside the CW-MFCs, both along the water flow direction and under different HRTs, are shown in Figure 2. It was observed that the NH_4_^+^-N concentration decreased sharply from layer U4 to layer D2, especially in CM-A. As the HRT decreased from 7.6 to 2.8 d, increased NH_4_^+^-N concentrations were observed in the sampling ports of the up-flow chamber of CM-A, the down-flow chamber of CM-B, and both chambers in CM-C. However, the CM-A effluent NH_4_^+^-N concentrations were mostly below 1.50 mg/L when the HRT decreased to 2.8 d. It is noteworthy that the effluent NO_x_^–^-N concentrations in all sampling ports of the CW-MFCs were mostly below 0.3 mg/L. The variation in PO_4_^3–^-P concentrations under different HRTs was similar to that in NH_4_^+^-N concentrations except for that of CM-B, demonstrating impressive PO_4_^3–^-P treatment efficiency in CM-A. According to Table 1, both the effluent NH_4_^+^-N and PO_4_^3–^-P concentrations in CM-A were significantly lower than those in CM-B and CM-C under different HRTs (*P* < 0.05).

**TABLE 1:** N, P, and COD concentrations in the influent and effluent of the CW-MFCs under different HRTs.

	**HRT**	**Influent**	**Effluent**		
			**CM-A**	**CM-B**	**CM-C**
NH_4_^+^-N (mg/L)	7.6 d	18.04 ± 2.75	0.75 ± 0.14^a,A^	11.54 ± 1.21^b,A^	6.43 ± 1.39^c,A^
	4.0 d	16.36 ± 1.20	0.28 ± 0.14^a,B^	15.27 ± 1.30^b,B^	11.94 ± 1.45^c,B^
	2.8 d	16.74 ± 1.25	1.03 ± 0.34^a,A^	16.68 ± 1.51^b,B^	14.65 ± 1.47^c,C^
NH_4_^+^-N removal efficiency (%)	7.6 d		95.8	36.1	64.4
	4.0 d		98.3	6.7	27.0
	2.8 d		93.8	0.4	12.5
PO_4_^3–^-P (mg/L)	7.6 d	7.00 ± 0.55	0.35 ± 0.19^a,A^	8.19 ± 0.81^b,A^	5.67 ± 0.23^c,A^
	4.0 d	7.29 ± 0.58	0.03 ± 0.01^a,B^	8.03 ± 0.26^b,A^	6.62 ± 0.28^c,B^
	2.8 d	7.24 ± 0.19	0.03 ± 0.01^a,B^	8.47 ± 0.17^b,A^	7.61 ± 0.19^c,C^
PO_4_^3–^-P removal efficiency (%)	7.6 d		95.0	−17.0	19.0
	4.0 d		99.6	−10.1	9.2
	2.8 d		99.6	−17.0	−5.1
COD (mg/L)	7.6 d	262 ± 69	9 ± 8^a,A^	11 ± 4^a,A^	15 ± 12^a,A^
	4.0 d	243 ± 22	90 ± 21^a,B^	18 ± 6^b,A,B^	87 ± 21^a,B^
	2.8 d	243 ± 15	128 ± 12^a,C^	20 ± 5^b,B^	94 ± 11^c,B^
COD removal efficiency (%)	7.6 d		96.6	95.7	94.2
	4.0 d		62.9	92.6	64.0
	2.8 d		47.3	91.8	61.3

*All data are presented as the means ± SD (standard deviation); *n* = 6 for each group. ^*a,b,c*^Indicate significant differences in effluent water quality among CM-A, CM-B, and CM-C. ^*A,B,C*^Indicate that there were significant differences among the systems operated under different HRTs.*

**FIGURE 3:**
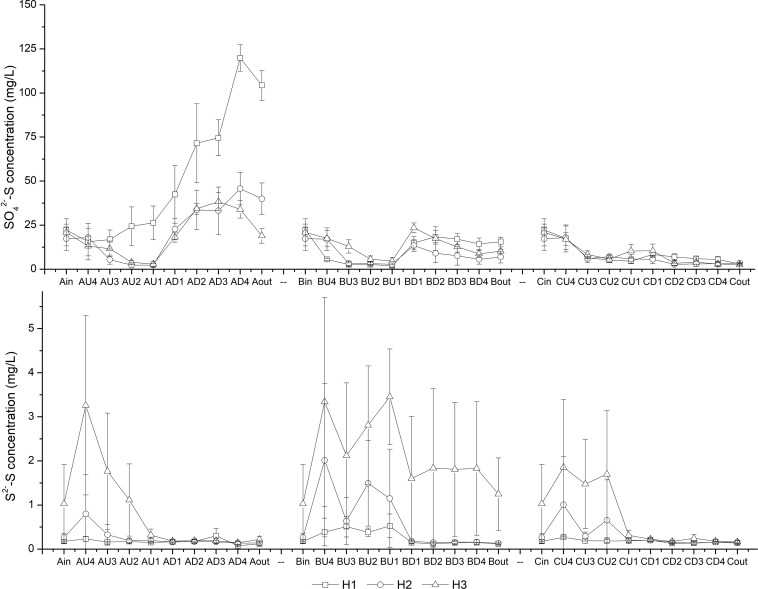
Variation in SO_4_^2–^-S and S^2–^-S concentrations at different layers [influent (in), U4-U1, D1-D4 and effluent (out)] of CW-MFCs filled with ceramsite (CM-A), quartz (CM-B), and zeolite (CM-C) granules under various hydraulic retention times [7.6 d (H1), 4.0 d (H2), and 2.8 d (H3)].

As the HRT decreased from H1 (7.6 d) to H3 (2.8 d), the COD concentrations in the effluent increased accordingly (Table 1). The lowest effluent COD concentrations in CM-B were observed among the three CW-MFCs under HRTs of 4.0 and 2.8 d. It is interesting to find that the effluent SO_4_^2–^-S concentrations in CM-A were higher than those in the influent, while the effluent S^2–^-S concentrations of the CW-MFCs were negligible except for those of CM-B (Figure 3).

With an HRT of 7.6 d (H1), the average voltage outputs in both the up-flow and down-flow chambers in CM-A were higher than those in CM-B and CM-C (Supplementary Figure S1). The average power densities in the up-flow chamber were 120.3, 11.3, and 14.2 mW/m^3^ in CM-A, CM-B, and CM-C, respectively. However, when the HRT was decreased to 4.0 d (H2) and 2.8 d (H3), low voltage outputs (below 0.02 V) with intermittent fluctuations in the three CW-MFCs were observed. Additionally, there were sharp decreases in voltage output in the up-flow chamber after the inflowing of synthetic wastewater.

### Bacterial Morphologies and Community Structures in the CW-MFCs

**FIGURE 4:**
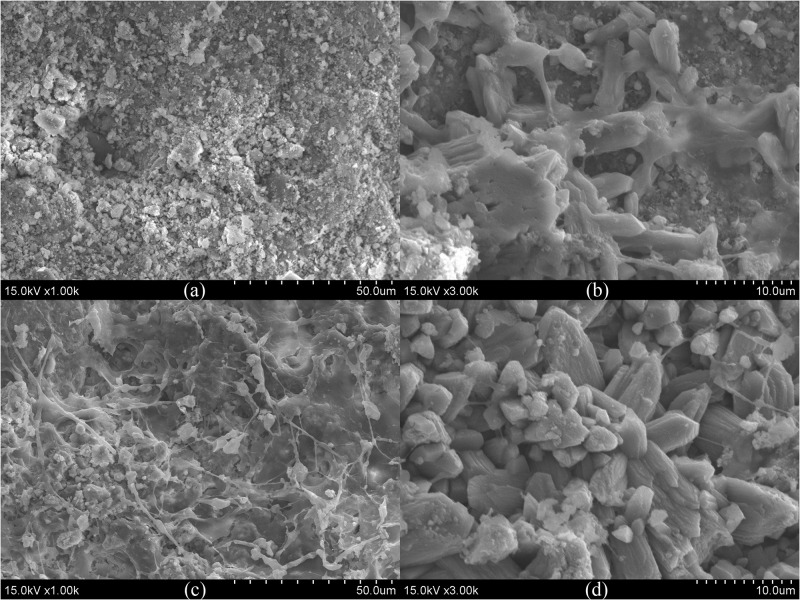
SEM images of the surface of ceramsite granules (**a**: original ceramsite granule, **b–d**: ceramsite granules at layers U4, D1 and D2 of CM-A).

The morphologies of the ceramsite granules at different layers were observed by SEM, and selected images are shown in Figure 4. Structures like mineral crystals were observed on the surface of ceramsite granule at layers U4 and D2. Via EDS analysis, an increase in calcium and a decrease in aluminum and silicon on the surface of the ceramsite granules were observed in layer AD2 compared with the original ones; and the P content on the surface of ceramsite granules sampled from layers AD1 and AD2 increased compared with the original ones (Supplementary Table S2). The crystalline mineral might be associated with Ca and P deposition. Additionally, the SEM images in Supplementary Figure S2 present morphology and structure of quartz and zeolite granules. Except for quartz granules, ceramsite and zeolite granules displayed porous structure and had large specific surface area. Both the SEM images and the results of EDS analysis (the variation in C and N contents) showed that the surface of substrates at layer D1 of the three CW-MFCs was covered by adhesive matrix, which was possibly excreted by the aggregated microorganisms.

To evaluate the potential influences of substrate selection on the bacterial groups involved in N and P removal in the cathode region, the bacterial communities close to the cathodes of the CW-MFCs were determined by 16S rRNA gene sequencing. The results of the richness indices, including OTU numbers and the ACE, Simpson, Sobs and Chao1 indices, are summarized in Supplementary Table S3. The Chao1 index values, which are used to estimate the richness of the total bacterial community, were 559.60 for CM-A, 663.17 for CM-B and 628.40 for CM-C. A similar order was observed in the values of indicators such as OTU numbers, ACE, and Sobs. These findings show that the samples from CM-B had the highest richness, followed by those from CM-C and CM-A. According to the values of Shannon index and Simpson index, the bacterial diversities were in the order of CM-B > CM-A > CM-C. Good’s coverage values of all the three samples were above 0.99.

**FIGURE 5:**
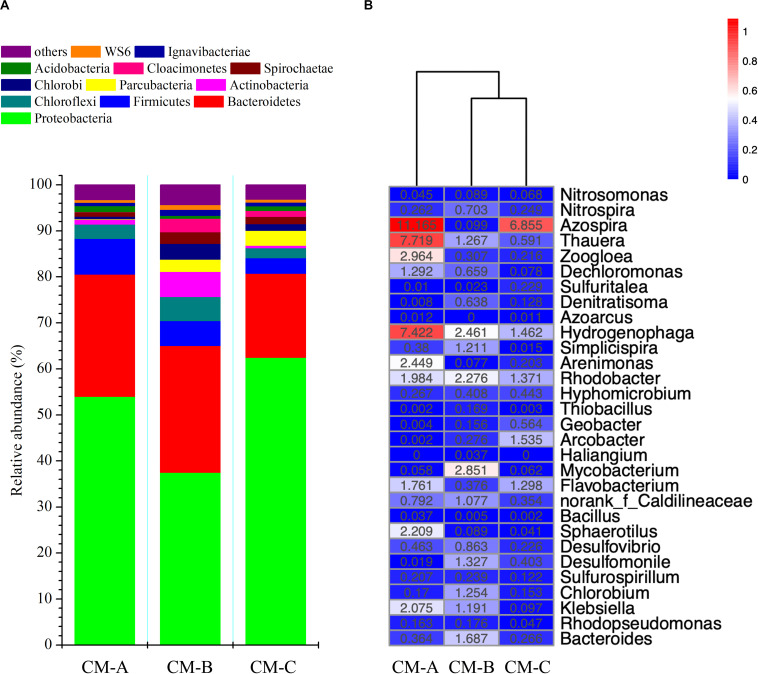
Bacterial community composition at the phylum level **(A)** and relative abundance of potential functional genera responsible for nitrogen and sulfur cycling and electricity generation **(B)** in the CW-MFCs. The color intensity in each panel indicates the similarity or difference in characteristics among CM-A, CM-B, and CM-C; red and blue colors represent low or high enrichment of a genus, respectively **(B)**.

According to the community barplot analysis at the phylum level (Figure 5A), the majority of the sequences belong to the phyla *Proteobacteria* and *Bacteroidetes*, comprising 80.6, 65.1, and 80.7% of the total sequences in CM-A, CM-B, and CM-C, respectively. Within *Proteobacteria*, the β-subdivision was the predominant group, followed by γ-*Proteobacteria*, α-*Proteobacteria*, δ-*Proteobacteria*, and ε-*Proteobacteria*. At the family level, *Comamonadaceae*, *Lentimicrobiaceae*, and *Rhodocyclaceae* were dominant families in the CW-MFCs. The dominant families accounted for 50.9, 33.0, and 42.8% of the classified sequences in CM-A, CM-B, and CM-C, respectively.

Relative abundances of bacterial taxa that are potentially related to nitrification, denitrification, SO_4_^2–^ reduction, and electricity generation are presented in Figure 5B. The genera *Nitrosomonas* and *Nitrospira* accounted for 0.3, 0.8, and 0.3% of the detected OTUs in CM-A, CM-B and CM-C, respectively. High abundance of potential denitrifying bacteria (e.g., *Azospira*, *Thauera*, *Hydrogenophaga*, *Zoogloea*, *Arenimonas*, *Rhodobacter*, *Flavobacterium*, and *Dechloromonas*) at the genus level were observed, accounting for 35.8, 14.3 and 15.2% of the detected OTUs in CM-A, CM-B, and CM-C, respectively. The majority of the above genera belong to the β-subclass of the *Proteobacteria*. The abundance of the genera *Desulfovibrio* and *Desulfomonile* was higher in CM-B (2.2%) than in CM-A (0.5%) and CM-C (0.6%). A slightly higher abundance of the genera *Klebsiella*, *Bacteroides*, *Rhodopseudomonas*, and *Arcobacter* was observed in CM-B (3.1%) than in CM-A (2.6%) and CM-C (0.4%). Notably, the highest abundance of the genus *Sphaerotilus* (a potential iron-oxidizing bacterium) was observed in CM-A (2.2%) compared with CM-B (0.1%) and CM-C (0.1%).

## Discussion

### Nitrogen Removal Pathways

From layer AU4 to AU2, it was unexpected that efficient NH_4_^+^-N removal was observed. In contrast, the effluent NH_4_^+^-N concentrations remained almost the same from layer BU4 to BU2 under all the three HRTs, while the effluent NH_4_^+^-N concentrations decreased from layer CU4 to CU2 with an HRT of 7.6 d. The effluent pH values in the up-flow chamber of CM-A (above 8.00) were higher than those in CM-B and CM-C under all the three HRTs (Supplementary Table S1). The relatively higher pH values in the up-flow chamber of CM-A could contribute to the ammonia loss via volatilization. In CM-C, the NH_4_^+^-N could be adsorbed by the zeolite granules under relatively long HRT (7.6 d). The results indicated that the NH_4_^+^-N removal from layer U4 to U2 might be associated with the substrates selected.

Although the average effluent DO concentrations (in the range of 2.03 to 3.60 mg/L) in the up-flow chamber of the three CW-MFCs under three HRTs were not so low as to inhibit nitrification (Supplementary Table S1), it was unexpected to notice low abundance of ammonium-oxidizing bacteria (i.e., *Nitrosomonas*) and nitrite-oxidizing bacteria (i.e., *Nitrospira*) in the cathode area of CW-MFCs. However, promising N removal efficiency was observed in CM-A without the assistance of extra energy-demanding measures (i.e., aeration and effluent recirculation). Especially in the cathode area (from layer U2 to D2) of CM-A, efficient NH_4_^+^-N removal and low effluent NO_x_^–^-N concentrations were observed as expected. The abundance of denitrifying bacteria in the cathode area in CM-A was the highest among the three CW-MFCs. Some of the denitrifying bacteria might serve versatile functions in the N removal process. Feng et al. (2015) indicated that certain groups of bacteria (e.g., *Hydrogenophaga*) were capable of heterotrophic nitrification and aerobic denitrification (HNAD) in the cathode chamber of an MFC. In a tidal flow CW, the genera *Hydrogenophaga*, *Dechloromonas*, and *Zoogloea* were recognized as HNAD bacteria, which were essential to achieve good N removal performance via simultaneous nitrification and denitrification (SND) process (Tan et al., 2020). In accordance with these studies, relatively higher abundance of *Hydrogenophaga*, *Dechloromonas*, and *Zoogloea* was observed in CM-A compared with that in CM-B and CM-C, which might contribute to the efficient N removal from layer AU2 to AD2 in this study. Moreover, Virdis et al. (2011) indicated that the SND process in the cathode chamber could be promoted in relatively high DO concentrations (5.73 ± 0.03 mg/L) if biofilm stratification (i.e., the nitrifying bacteria appear in the outer layer of the biofilm and the putative denitrifying organisms occupy the inner layer) was achieved. According to the SEM images (Figure 4C) and the composition of bacterial community, the SND process in the cathode area of CM-A could possibly benefit from the formation of biofilm on the surface of the ceramsite granules.

In the up-flow chamber of the CW-MFCs, the SO_4_^2–^-S concentrations decreased from layers U4 to U2 at most times, while the S^2–^-S concentrations increased simultaneously compared with those in the influent (Figure 3). In the down-flow chamber of the CW-MFCs, the S^2–^-S was consumed from layers U2 to D1, suggesting the S^2–^-S might be involved in the denitrifying sulfide removal (DSR) process. Huang et al. (2017) stated that not only autotrophic bacteria can perform the DSR process, but also some heterotrophic bacteria are capable of DSR, achieving the simultaneous removal of NOx^–^-N, S^2–^-S and organic carbon. *Thauera* was recognized as a sulfide-tolerant and facultative denitrifier (Mao et al., 2013; Liang et al., 2020), and it became the predominant bacterial genus when both sulfide and organic carbon were provided (Zhang et al., 2019). Liu et al. (2015) reported *Thauera* as one of the key strains in a denitrifying ammonium oxidation reactor treating organics-deficient wastewater with excess S^0^ production. In this study, relatively high abundance of genus *Thauera* in CM-A (7.72%) might also play a vital role in efficient N removal. In CM-B, the dominance of sulfate-reducing bacteria (e.g., *Desulfovibrio* and *Desulfomonile*) in the up-flow chamber might contribute to the increased COD removal efficiency and S^2–^ accumulation when the HRT decreased to 2.8 d. Due to the low NH_4_^+^-N removal rate and nitrate production rate, the S^2–^ consumption by DSR process would be decreased, which might be the reason why the highest effluent S^2–^ concentration was observed in CM-B.

Moreover, the results of bacterial community analysis indicate that autotrophic denitrification using ferrous iron as electron donor might be involved in N removal process in the cathode area of CM-A. *Sphaerotilus* strains have the ability to deposit insoluble oxides or hydroxides of iron on their sheaths, providing resistance to growth inhibition caused by high ferric concentrations (Rogers and Anderson, 1976). In this study, relatively higher abundance of *Sphaerotilus* was observed in CM-A (2.21%) compared with that in CM-B (0.09%) and in CM-C (0.04%). The substrates filled in the CW-MFCs were identical to those used in Zhong et al. (2015), in which iron (Fe) content in the milled ceramsite, quartz, and zeolite granules were 41,791 ± 452 mg/kg, 8,096 ± 111 mg/kg, and 219 ± 19 mg/kg, respectively. The high Fe content in ceramsite granules might encourage the growth of *Sphaerotilus* in CM-A. The oxidation of ferrous ions and reduction of ferric ions, which are carried out by the Fe transformation functional bacterial groups, may have synergetic relationships with denitrifiers and contribute to denitrification (Zhang et al., 2018). Li et al. (2016) reported that *Azospira*, *Zoogloea*, and *Dechloromonas* played important roles in both the nitrate reduction and ferrous ions oxidation in the soil suspension. In CM-A, the relative abundances of *Azospira*, *Zoogloea*, and *Dechloromonas* were 11.17, 2.96, and 1.29%, respectively. Their dominance in CM-A would be beneficial to the denitrification process by donating electrons via oxidizing ferrous ions to ferric ions. Although the variations in ferric and ferrous concentrations were not measured in this study, it could be inferred that the efficient N removal in CM-A might be partly ascribed to the high Fe content in ceramsite granules and the internal transformation and cycling of ferric and ferrous ions.

The co-existence of autotrophic and heterotrophic denitrification processes had been reported in wastewater treatment systems under different influent COD/N ratios (Xu D. et al., 2017). Some genera (e.g., *Zoogloea*) are capable of both autotrophic and heterotrophic denitrification (Su et al., 2020), which might play a vital role under mixotrophic conditions. Additionally, the synthetic wastewater with relatively high COD/N ratio was first pumped into the anode area of the up-flow chamber in this study. The organic compounds would be partly consumed before arriving at the cathode area as reported (Oon et al., 2017; Yakar et al., 2018). The impact of organic compounds on the activity of the autotrophic denitrifiers would be limited. Xu D. et al. (2017) investigated the effects of COD/N on the simultaneous heterotrophic and hydrogen-based autotrophic denitrification performance in a bioelectrochemically assisted CW, and their study found that the N removal efficiency were significantly increased with increasing COD/N ratio. The combined heterotrophic and autotrophic denitrification processes could contribute to the relatively high and stable N removal performance in the cathode area of CM-A in this study.

### Phosphorous Removal Pathways

Compared with quartz and zeolite granules, Zhong et al. (2015) recommended ceramsite granules as the best substrate for efficient P removal in a horizontal subsurface flow CW. The substrates filled in the CW-MFC were the same as those reported by Zhong et al. (2015), and the high calcium and Fe content and the heating process used to produce porous ceramsite granules could also be the reasons for the highest PO_4_^3–^-P removal efficiencies in CM-A. The EDS analysis confirmed that the P content on the surface of ceramsite granules at layers AD1 and AD2 increased compared with the original ones. It has been reported that PO_4_^3–^-P can be removed by metal oxides adsorbents, and the occurrence of ion exchange between SO_4_^2–^-S and PO_4_^3–^-P was observed (Wu et al., 2014). The increased effluent SO_4_^2–^-S concentrations in CM-A could be partly related to the efficient removal of PO_4_^3–^-P by the ceramsite granules.

Biological P removal might also contribute to the efficient PO_4_^3–^-P removal in CM-A. Wu et al. (2013) reported that a synergistic relationship may exist between sulfur cycle and biological P removal. In the sulfur cycle-associated enhanced biological P removal (EBPR) process, poly-S served as one of the energy sources for P uptake and poly-P synthesis and ended up as sulfate; the genus *Thauera* was regarded as a key organism in the sulfur cycle-associated EBPR process and were assumed to participate in the denitrifying P and S^2–^-S removal from sulfate-rich wastewater (Guo et al., 2019). Additionally, the genus *Dechloromonas* has been reported as denitrifying poly-P accumulating organism that is capable of denitrifying P removal (Yuan et al., 2016). In this study, relatively high abundance of *Thauera* and *Dechloromonas* and SO_4_^2–^-S concentrations were observed in CM-A, suggesting denitrifying P removal might take place in this type of CW-MFC filled with ceramsite granules.

### Electricity Generation

The genera *Klebsiella*, *Arcobacter*, *Bacteroides*, and *Rhodopseudomonas* have been widely accepted as potential electrochemically active bacteria in the anode (Xia et al., 2010; Huang et al., 2014; Xiao et al., 2015). In this study, their appearance in the cathode area was observed, and only the abundance of *Klebsiella* was higher in CM-A compared with that in CM-B and CM-C. Whether or not they could be still electrochemically active in the cathode area deserve further investigation.

Additionally, *Thauera* was recognized as the major genus on an autotrophic denitrifying biocathode (Jiang et al., 2017). Yang et al. (2018) reported that selected *Thauera*-dominated cultures could achieve high performance in electricity generation in parallel with N removal. Besides involved in N removal, *Rhodobacter* and *Hydrogenophaga* were also thought to play a role in electron transfer from the cathode surface to the terminal electron acceptor (Huang et al., 2014). The predominance of *Thauera*, *Hydrogenophaga*, and *Rhodobacter* in the cathode area of CM-A might contribute to the enhancement of voltage output in CM-A compared with CM-B and CM-C.

Due to the low voltage output of the CW-MFCs under HRTs of 4.0 and 2.8 d, power density was not presented. During the experiment, the influent was pumped into the CW-MFCs in a short time. When the HRT was decreased, the influent wastewater with low DO concentrations might flow to the cathode area directly, resulting in the reduction in power output of CW-MFCs. While taking efforts to increase voltage output of this system, future studies should probe other approaches that can make full use of the generated electricity.

Output bioelectrical signals (e.g., voltage) of the CW-MFCs have been reported to correspond to changes in COD concentration (Xu L. et al., 2017; Corbella et al., 2019). In this study, it is noteworthy that the voltage output of the CW-MFCs was intermittently fluctuated. As the daily variation in voltage output was highly related to the time of pumping in this study (Supplementary Figure S1), the signals could be used to sense wastewater inflow. If the wastewater inflow was blocked due to clogging problems, it could be expected that the regular fluctuation in output voltage will change. Further studies should be conducted to examine whether bioelectrical signals could be regarded as an indicator of system clogging problems. If so, the decrease in nutrient removal efficiency induced by clogging problems could be timely detected via the abnormal fluctuation of bioelectrical signals.

## Conclusion

CW-MFCs consisting of up-flow and down-flow chambers could remove NH_4_^+^-N and PO_4_^3–^-P efficiently when filled with ceramsite granules. The N and P removal efficiencies in the CW-MFCs filled with ceramsite granules were relatively stable under different HRTs, while the voltage outputs were greatly reduced when the HRTs decreased from 7.6 to 2.8 d. The efficient N removal in CM-A could be ascribed to the appearance of nitrifying bacteria (e.g., *Nitrosomonas* and *Nitrospira*) and relatively high abundances of denitrifying bacteria in the cathode area that were potentially involved in SND (e.g., *Hydrogenophaga*, *Zoogloea*, and *Dechloromonas*), DSR (e.g., *Thauera*) and ferrous ion-dependent nitrate removal (e.g., *Azospira*, *Zoogloea*, and *Dechloromonas*). Substrate adsorption and denitrifying P removal via the genera *Thauera* and *Dechloromonas* might be the dominant pathways of P removal. Concerning the impressive purification performance, ceramsite granules are recommended rather than quartz and zeolite granules as substrates for the integrated vertical-flow CW-MFCs.

## Data Availability Statement

We have deposited the data in NCBI with accession number PRJNA629887.

## Author Contributions

FZ: idea. FZ, CY, YC, JW, JZ, and ZD: data analysis. CY, YC, XW, and GL: data collection. FZ and SC: writing and reviewing the manuscript. All authors contributed to the article and approved the submitted version.

## Conflict of Interest

The authors declare that the research was conducted in the absence of any commercial or financial relationships that could be construed as a potential conflict of interest.

## Footnotes

1 http://drive5.com/uparse/

2 http://rdp.cme.msu.edu/
