# Supplementary material for: Nutrient Removal Process and Cathodic Microbial Community Composition in Integrated Vertical-Flow Constructed Wetland – Microbial Fuel Cells Filled With Different Substrates
Source: Front Microbiol. 2020 Aug 5;11:1896. doi: 10.3389/fmicb.2020.01896 (PMC7419476; doi:10.3389/fmicb.2020.01896)
Supplement: Supplementary file 1 [file Data_Sheet_1.DOCX]

**Supplementary materials**

**Nutrient Removal Process and Cathodic Microbial Community Composition in Integrated Vertical-flow Constructed Wetland – Microbial Fuel Cells Filled with Different Substrates**

Fei Zhong^*1^, Chunmei Yu^1^, Yanhong Chen^1^, Xue Wu^2^, Juan Wu^2,3^, Guoyuan Liu^1^, Jian Zhang^1^, Zifa Deng^1^, Shuiping Cheng^*2,3^

1. School of Life Sciences, Nantong University, Nantong 206019, PR China

2. Key Laboratory of Yangtze River Water Environment, Ministry of Education, College of Environmental Science and Engineering, Tongji University, Shanghai 200092, PR China

3. Shanghai Institute of Pollution Control and Ecological Security, Shanghai, 200092, PR China

^*^Corresponding author:

Fei Zhong, Tel.: +86 513 85012825. E-mail address: [fzhong@ntu.edu.cn](mailto:fzhong@ntu.edu.cn);

Shuiping Cheng, Tel.: +86 21 65980763. E-mail address: shpcheng@tongji.edu.cn


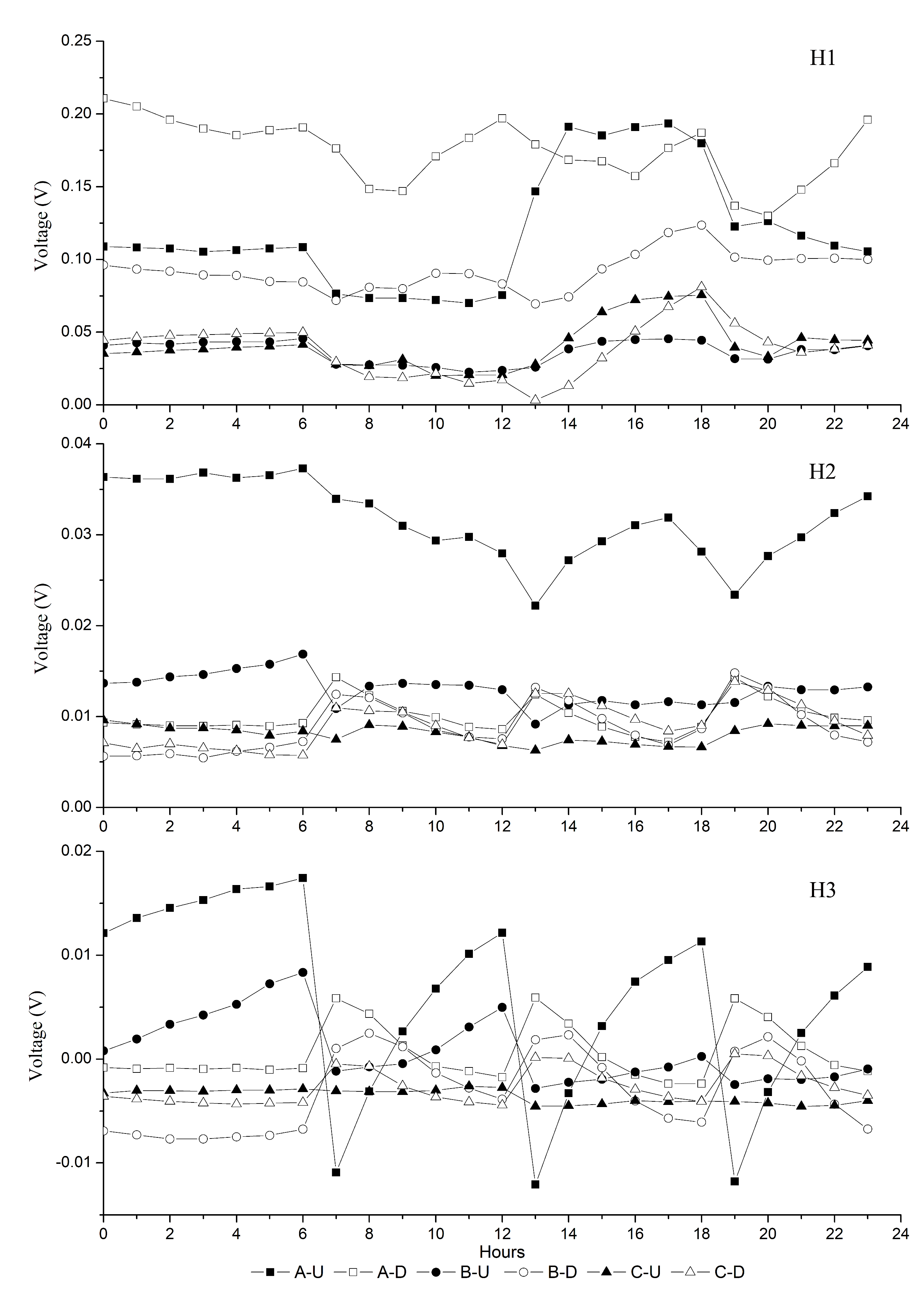


**Fig S1.** Daily variation in voltage output in the up-flow chamber (U) and down-flow chamber (D) of CW-MFCs filled with ceramsite (CM-A), quartz (CM-B), and zeolite (CM-C) granules under various hydraulic retention times (7.6 d (H1), 4.0 d (H2), and 2.8 d (H3))

**
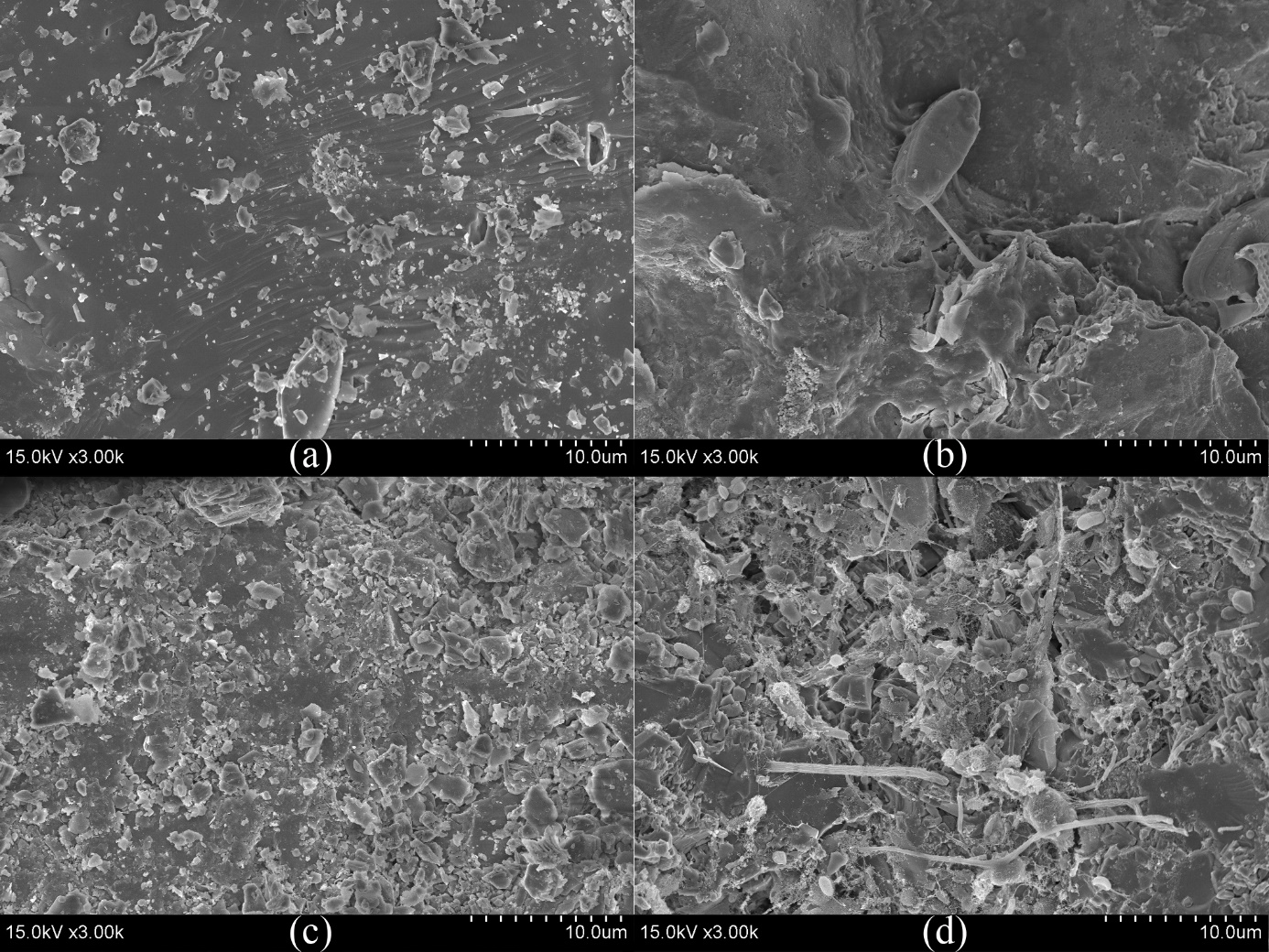
**

**Fig S2.** SEM images of the surface of quartz and zeolite granules (a, c: original quartz and zeolite granules, respectively; b, d: quartz and zeolite granules at layer D1 of CM-B and CM-C, respectively)

**Table S1** pH and DO values in the influent and effluent of the CW-MFCs under different HRTs.

|  | HRT | Influent | CM-A effluent | | CM-B effluent | | CM-C effluent | |
| --- | --- | --- | --- | --- | --- | --- | --- | --- |
|  |  |  | Up-flow chamber | Down-flow chamber | Up-flow chamber | Down-flow chamber | Up-flow chamber | Down-flow chamber |
| pH | 7.6 d | 6.71 ± 0.25 | 8.01 ± 0.09 | 9.12 ± 0.13 | 7.72 ± 0.08 | 7.81 ± 0.12 | 7.59 ± 0.15 | 7.71 ± 0.13 |
|  | 4.0 d | 7.50 ± 0.49 | 8.38 ± 0.24 | 8.97 ± 0.14 | 7.45 ± 0.11 | 7.60 ± 0.05 | 7.33 ± 0.08 | 7.51 ± 0.10 |
|  | 2.8 d | 6.88 ± 0.05 | 8.36 ± 0.21 | 8.17 ± 0.02 | 7.34 ± 0.07 | 7.45 ± 0.04 | 7.25 ± 0.06 | 7.34 ± 0.05 |
| DO (mg/L) | 7.6 d | 0.10 ± 0.03 | 3.60 ± 0.66 | 4.29 ± 0.80 | 2.12 ± 0.92 | 4.45 ± 0.78 | 2.92 ± 0.87 | 4.91 ± 0.83 |
|  | 4.0 d | 0.24 ± 0.22 | 3.40 ± 0.79 | 3.72 ± 0.53 | 2.24 ± 0.31 | 3.66 ± 0.74 | 3.28 ± 0.08 | 3.23 ± 0.92 |
|  | 2.8 d | 0.47 ± 0.30 | 2.63 ± 0.69 | 3.02 ± 0.64 | 2.03 ± 1.13 | 2.23 ± 0.15 | 2.21 ± 1.05 | 3.31 ± 0.66 |

All data are presented as the means ± SD (standard deviation); n=6 for each group.

**Table S2** EDS analysis of the substrates before (Original) and after (at layer AD1, AD2, BD1 and CD1 of the CW-MFCs) use.

| Elements (wt%) | | C | O | Na | Mg | Al | Si | S | K | Ca | Fe | P | N | Total |
| --- | --- | --- | --- | --- | --- | --- | --- | --- | --- | --- | --- | --- | --- | --- |
| Ceramsite | Original | 22.33 | 50.02 | 0.35 | 0.55 | 6.15 | 8.33 | 0.89 | 0.79 | 9.60 | 1.01 | 0.00 | 0.00 | 100.00 |
|  | AD1 | 32.90 | 36.50 | 0.00 | 1.40 | 4.60 | 8.50 | 0.80 | 0.00 | 8.10 | 2.00 | 0.60 | 4.60 | 100.00 |
|  | AD2 | 17.75 | 48.42 | 0.00 | 0.43 | 3.64 | 5.46 | 0.48 | 0.56 | 20.30 | 2.06 | 0.80 | 0.00 | 100.00 |
| Quartz | Original | 3.07 | 48.53 | 0.00 | 0.10 | 1.60 | 45.73 | 0.20 | 0.00 | 0.00 | 0.00 | 0.00 | 0.80 | 100.00 |
|  | BD1 | 8.40 | 49.80 | 0.00 | 0.10 | 0.00 | 35.20 | 0.30 | 0.00 | 3.30 | 0.00 | 0.00 | 3.00 | 100.00 |
| Zeolite | Original | 4.30 | 53.77 | 0.00 | 0.00 | 12.43 | 28.30 | 0.00 | 0.00 | 0.00 | 0.00 | 0.00 | 1.20 | 100.00 |
|  | CD1 | 8.90 | 44.90 | 0.00 | 0.20 | 6.70 | 28.60 | 0.30 | 2.90 | 2.50 | 2.20 | 0.00 | 2.70 | 100.00 |

**Table S3** Diversity indices of the microbial communities around the cathodes of the CW-MFCs

|  | Operational taxonomic unit | Sobs | Shannon | Simpson | Ace | Chao | coverage |
| --- | --- | --- | --- | --- | --- | --- | --- |
| CM-A | 51615 | 502 | 4.15 | 0.039 | 541.28 | 559.60 | 0.9988 |
| CM-B | 64899 | 642 | 4.72 | 0.032 | 659.65 | 663.17 | 0.9994 |
| CM-C | 61565 | 592 | 3.64 | 0.097 | 623.11 | 628.40 | 0.9991 |
